# The ICN-UN Battery: A Machine Learning-Optimized Tool for Expeditious Alzheimer’s Disease Diagnosis

**DOI:** 10.3390/diagnostics15233045

**Published:** 2025-11-28

**Authors:** Ernesto Barceló, Duban Romero, Ricardo Allegri, Eliana Meza, María I. Mosquera-Heredia, Oscar M. Vidal, Carlos Silvera-Redondo, Mauricio Arcos-Burgos, Pilar Garavito-Galofre, Jorge I. Vélez

**Affiliations:** 1 Instituto Colombiano de Neuropedagogía, Barranquilla 080020, Colombia; 2 Department of Health Sciences, Universidad de La Costa, Barranquilla 080002, Colombia; 3 Grupo Internacional de Investigación Neuro-Conductual (GIINCO), Universidad de La Costa, Barranquilla 080002, Colombia; 4 Institute for Neurological Research FLENI, Montañeses 2325, Buenos Aires C1428AQK, Argentina; 5 Department of Medicine, Universidad del Norte, Barranquilla 081007, Colombia; 6 Grupo de Investigación en Psiquiatría (GIPSI), Departamento de Psiquiatría, Instituto de Investigaciones Médicas, Facultad de Medicina, Universidad de Antioquia, Medellín 050010, Colombia; 7 Department of Industrial Engineering, Universidad del Norte, Barranquilla 081007, Colombia

**Keywords:** Alzheimer’s disease, Machine Learning, neuropsychological assessment, diagnostic protocol, cognitive impairment

## Abstract

**Background/Objectives**: Alzheimer’s disease (AD) accounts for ~70% of global dementia cases, with projections estimating 139 million affected individuals by 2050. This increasing burden highlights the urgent need for accessible, cost-effective diagnostic tools, particularly in low- and middle-income countries (LMICs). Traditional neuropsychological assessments, while effective, are resource-intensive and time-consuming. **Methods**: A total of 760 older adults (394 [51.8%] with AD) were recruited and neuropsychologically evaluated at the Instituto Colombiano de Neuropedagogía (ICN) in collaboration with Universidad del Norte (UN), Barranquilla. Machine learning (ML) algorithms were trained on a screening protocol incorporating demographic data and neuropsychological measures assessing memory, language, executive function, and praxis. Model performance was determined using 10-fold cross-validation. Variable importance analyses identified key predictors to develop optimized, abbreviated ML-based protocols. Metrics of compactness, cohesion, and separation further quantified diagnostic differentiation performance. **Results**: The eXtreme Gradient Boosting (xgbTree) algorithm achieved the highest diagnostic accuracy (91%) with the full protocol. Five ML-optimized screening protocols were also developed. The most efficient, the ICN-UN battery (including MMSE, Rey–Osterrieth Complex Figure recall, Rey Auditory Verbal Learning, Lawton & Brody Scale, and FAST), maintained strong diagnostic performance while reducing screening time from over four hours to under 25 min. **Conclusions**: The ML-optimized ICN-UN protocol offers a rapid, accurate, and scalable AD screening solution for LMICs. While promising for clinical adoption and earlier detection, further validation in diverse populations is recommended.

## 1. Introduction

Alzheimer’s Disease (AD) accounts for 70% of dementia cases and represents a growing global concern [1]. The disease is a significant burden on healthcare systems and millions of families worldwide. The number of affected individuals is expected to reach 78 million cases by 2030 and 139 million by 2050 [2].

With more than 60% of dementia patients residing in low- and middle-income countries (LMICs) [3], there is an urgent need for rapid and affordable diagnostic methods that enable early detection of AD across diverse socioeconomic groups, ensuring no one is excluded [4].

Traditional neuropsychological assessments are effective but are often resource-intensive and time-consuming. Variability in neuropsychological test administration, the influence of comorbidities, and possible confounding with vascular dementia. Nevertheless, neuropsychological variables remain cost-effective and non-invasive predictive biomarkers for AD diagnosis, especially where advanced imaging is inaccessible [5].

Clinical practitioners and researchers integrate a range of neuropsychological tests for evaluating neuropsychiatric pathologies. These include tools such as the Mini-Mental State Examination, Addenbrooke’s Cognitive Examination, Corsi’s Test, Trail Making Test, Symbol Digit Modalities Test, Tower of London-Drexel Version, Boston Naming Test, Rey–Osterrieth Complex Figure, Visual Object and Space Perception Battery, Clock Test, Wechsler Memory Scale, Digit Span, AD Assessment Scale-Cognitive Behavior, and the Functional Assessment Questionnaire [6,7,8,9]. While some of these tests achieve >85% accuracy for the diagnosis of AD [10,11,12], their combined administration is often lengthy and costly [13,14,15], restricting the number of participants in AD research.

Recently, Machine Learning (ML)-based predictive models have demonstrated value for diagnosis and monitoring in psychiatric and neurodegenerative disorders, including AD [16], bipolar disorder [17], Parkinson’s disease [18] and attention deficit hyperactivity disorder [19]. These models can identify patterns and achieve high diagnostic accuracy in relatively short timeframes. ML applications integrating multimodal data often outperform traditional methods, offering improved accuracy and interpretability [20]. ML-based models have also been extensively applied across various scientific domains, demonstrating remarkable effectiveness in addressing challenges such as digital image processing and financial forecasting [21,22].

Recent studies have applied ML-based models (i.e., Naïve Bayes, *K*-Nearest Neighbors, Gradient Boosting, Random Forest [RF], Support Vector Machines [SVMs], and Logistic Regression) to accelerate AD diagnosis in individuals exhibiting cognitive impairment. These models have achieved correct classification rates exceeding 85% [8,23,24].

Explainability of ML models is increasingly recognized as a critical aspect of ML in healthcare [25]. Techniques such as Shapley Additive Explanations (SHAP) [26] and Partial Dependence Plots (PDPs) [27] provide valuable insights into feature importance, helping clinicians discern which cognitive domains most strongly influence predictions. This transparency supports decision-making and enhances trust in predictive tools [28,29,30].

The present study aims to develop a reduced assessment protocol to optimize the time of neuropsychological evaluation of individuals diagnosed with AD through the implementation of ML-based models, identifying the neuropsychological tests that most contribute to achieving high detection rates of patients with AD.

The remainder of this manuscript is structured as follows. In Section 2, we detail the study design, participant selection, the neuropsychological assessment protocols, and the ML methods and feature selection procedures used to optimize the battery for AD diagnosis. Section 3 reports the main results, including the diagnostic performance of the reduced battery. Section 4 discusses the implications, limitations, and future directions. Finally, Section 5 provides our conclusions.

## 2. Subjects and Methods

### 2.1. Subjects

We included 760 individuals (394 AD cases and 366 healthy controls) over 65 years old from the Alzheimer’s disease SGR study [31] led by Universidad del Norte (UN), Barranquilla. This initiative is dedicated to unraveling the genetic landscape associated with AD susceptibility, age of onset, and disease progression in individuals from the Atlántico Department, located in the Northern Caribbean coast of Colombia.

As part of this collaborative effort, individuals were recruited at the Instituto Colombiano de Neuropedagogía (ICN) in Barranquilla, Colombia. The ICN team determined candidates’ eligibility based on the Montreal Cognitive Assessment (MoCA) results [16] and the inclusion criteria described elsewhere [32,33]. After eligibility was confirmed, data from clinical evaluations, family histories, comprehensive neurological and neuropsychological clinical examinations, structured interviews and genetic testing were collected.

### 2.2. Neuropsychological Assessment

As part of our comprehensive clinical evaluation protocol, all individuals completed an extensive battery of conventional neuropsychological tests covering multiple domains, including general cognitive function, memory, language, and daily living skills.

In addition to collecting sociodemographic data, this protocol included detailed neuropsychological assessments. The following tests were included: Mini-Mental State Examination, Mental Control–Wechsler Memory Scale, Semantic Verbal Fluency, Phonological Verbal Fluency, Rey–Osterrieth Complex Figure Test (ROCFT), Token Test–Short Version, Rey Auditory Verbal Learning Test (RAVLT), Boston Naming Test (BNT), Stroop Color and Word Test, Trail Making Test (TMT), Symbol Digit Test (SDT), Continuous Auditory Performance Test, Benton Visual Retention Test, Clock Drawing Test, Wisconsin Card Sorting Test (WCST), Memory Disorder Scale, Yesavage Depression Scale, Barthel Index, Lawton and Brody Scale, Katz Index, and the Functional Assessment Staging (FAST).

### 2.3. ML-Based Screening Tool for AD Diagnosis

#### 2.3.1. Preprocessing

Missing data, a common problem in clinical studies, was imputed using the methods implemented in the missForest package version 1.6.1 [34] of R version 4.5.0 [35]. missForest implements a data imputation method based on the RF algorithm, where initial values are assigned to missing data, typically using the mean or mode. An RF model is then constructed to predict the missing values for each variable, using the other variables as predictors. The imputed values are updated with the predictions generated by the model. This process is repeated iteratively, improving the predictions with each iteration, until a convergence criterion is met, or a maximum number of iterations is reached. The overall proportion of missing data is 4.78% across all registers. In this study, imputation was performed separately for training and testing. At no point was the outcome variable (diagnosis) included in the imputation process, ensuring that information leakage did not bias the performance of the classification models.

#### 2.3.2. ML Algorithms

We used the caret package version 7.0-1 [36,37] in R to construct predictive models of AD status (0: control; 1: case) including the neuropsychological measures and demographic variables (i.e., age at the beginning of the study, sex and years of education) as predictors. A 10-fold cross-validation procedure was employed to assess how the models will likely perform on future unseen data. To develop ML models for AD, we explored several algorithms, including: Generalized Linear Model (glm), *K*-Nearest Neighbors (knn), Recursive Partitioning and Regression Trees (rpart, rpart1se, rpart2), Bagged Classification Trees (treebag), SVMs with Linear, Radial and Polynomial Kernels (svmlinear, svmlinear2, svmradial and svmpoly, respectively), Random Forest (rf), Linear Discriminant Analysis (lda2), High Dimensional Discriminant Analysis (hdda), Gradient Boosting Machine (gbm), Extreme Gradient Boosting (xgbtree and xgblinear), Bayesian Generalized Linear Model (bayesglm), and Artificial Neural Networks (avNNet). These algorithms were selected for their capacity to manage complex relationships in the data and provide robust predictions. For details on these algorithms and their parameters, see Table S1 of the Supplementary Material.

The dataset (*n* = 760) was partitioned into training (70%, *n* = 533) and testing (30%, *n* = 227) datasets, ensuring equal proportions of AD cases and healthy controls in these partitions. Imputation was conducted separately in these data partitions. Hyperparameter tuning of ML algorithms was performed using grid-search in the training data following the implementation of the train() function in caret.

The performance of each ML algorithm was evaluated using accuracy metrics derived from the cross-validation process [38,39]. Each ML-based model was evaluated using several performance metrics for binary classifiers, including the accuracy, the Receiver Operating Characteristic (ROC) curve, and the area under the ROC curve (AUC), sensitivity, specificity, precision, lift, negative predictive value and positive predictive value. These metrics assess how well the model predictions align with actual outcomes.

### 2.4. Construction of Neuropsychological Protocols

We optimized four neuropsychological protocols for AD screening using ML. Protocol #1 incorporated all neuropsychological variables in addition to demographic predictors including sex, age, and years of education as predictors. The ML algorithm with the highest diagnostic accuracy in the training dataset was retained for further analysis. Variable importance was then evaluated with this model to rank the contribution of each neuropsychological measure, and the five most informative variables were identified. These five top predictors formed the basis of Protocol 2, the ICN-UN Neuropsychological Battery.

Protocol #3 was developed using the one-rule (OneR) ML, implemented through the OneR package version 2.2 [40] in R. This algorithm creates one-rule models for each predictor in the dataset and selects the single most predictive attribute for the outcome variable of interest [41]. In this case, neuropsychological variables served as predictors of AD diagnosis (0: control; 1: case). The protocol included the top five neuropsychological variables with the highest accuracy for AD diagnosis identified using OneR.

Protocol #4 was developed by calculating the Predictive Power Score (PPS) [42] for each neuropsychological variable to assess their relevance in AD diagnosis. The PPS quantifies the strength and quality of predictive relationships, capturing both non-linear and asymmetric associations, making it particularly useful for identifying key variables in complex predictive modeling. We used the ppsr package version 0.0.5 (van der Laken, 2024 [42]) of R to compute these scores. The protocol incorporated the five neuropsychological variables with the highest PPS for discriminating AD. Additionally, given the diagnostic value of the BNT and RAVLT for AD diagnosis [10,11,12], we also developed separate protocols assessing the contribution of each of these tests.

#### 2.4.1. Compactness, Cohesion and Separation of Neuropsychological Protocols

We assessed the compactness of individuals diagnosed with AD and healthy controls for each protocol by calculating the compactness coefficient using the diceR [43,44] package version 3.1.0 of R. This internal validation index quantifies cluster quality based on the compactness (cohesion within clusters) and separability (distinctness between clusters) between groups; lower values indicate a better-defined clustering structure. To further evaluate the protocols, we computed the silhouette coefficient using the silhouette() function from the cluster [45] package version 2.1.8.1 of R. The silhouette coefficient ranges from −1 to +1, with higher values indicating greater cohesion within clusters and clearer separation from other groups [45,46]. Values near 0 suggest that the individual lies near the boundary between clusters, and negative values imply potential misclassification.

#### 2.4.2. Explainability of ML-Based Predictions

Partial dependence plots (PDPs) [27] help interpret complex black-box models by revealing the nature of the relationship between features and the target variable, including linearity, monotonicity, or more intricate patterns. Using the pdp package version 0.8.2 [47,48] in R, we generated PDPs to visualize the marginal effect of one or two features on the model’s predicted outcome while averaging over the remaining features.

## 3. Results

### 3.1. Participants

We collected data from 760 individuals (477 [63.2%] female; 394 [51.8%] diagnosed with AD) through clinical evaluations, family histories, comprehensive neurological and neuropsychological clinical examinations, and structured interviews. The Universidad del Norte Ethics Committee approved this study (Project Approval Act #198 of 31 October 2019). Table 1 summarizes the demographic characteristics of our sample.

We found statistically significant differences in the average age at the time of inclusion, with AD individuals up to 5 years older than healthy controls (AD: 78.9 ± 7.51; Controls: 74.29 ± 6.41, *p* < 0.001; Table 1), and in the gender distribution ($\chi_{1}^{2}=4$.84, *p* = 0.027; Table 1). Interestingly, 67% of the patients with AD were women, compared to 59% in the control group. However, no statistically significant difference was identified in the average years of education (schooling) between the comparison groups (AD: 8.85 ± 14.67; Controls: 9.21 ± 13.48; *p* = 0.723; Table 1).

Table 2 presents the results of comparing the neuropsychological tests between the groups included in this study. Several tests showed statistically significant differences between groups, indicating their potential relevance for distinguishing individuals with an AD diagnosis in a predictive model.

### 3.2. ML-Optimized Protocols for AD Diagnosis

**Figure 1:**
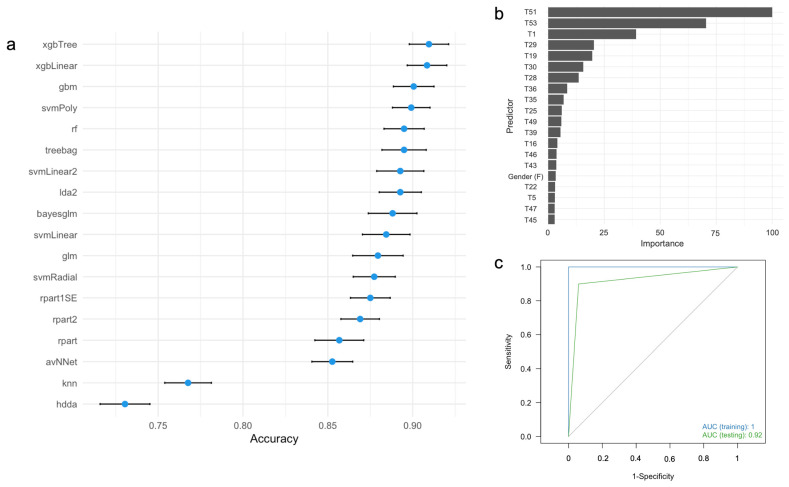
(**a**) Accuracy and 95% confidence intervals for predicting AD diagnosis using different ML algorithms based on full neuropsychological protocol. (**b**) Variable importance analysis for the xgbTree algorithm. (**c**) ROC curves for the xgbTree algorithm in the training (blue) and testing (green) datasets. ROC, receiver operating characteristic; AUC, area under the ROC curve. For the labels of the predictors, see Table 2.

We evaluated the performance of 18 ML algorithms to construct a predictive framework for AD diagnosis based on the demographic variables and the full neuropsychological protocol (that is, Protocol #1). The main results of this approach are presented in Figure 1.

In the training dataset, the xgbTree, xgLinear, and gbm algorithms demonstrated the best performance, while hdda and knn showed the lowest (Figure 1a). Variable importance analysis of the xgbTree algorithm identified the five most influential neuropsychological measures as T51 (Lawton and Brody test), T53 (FAST), T1 (MMSE), T29 (Rey Auditory Verbal Memory Recognition, Yes), and T19 (Recall of the ROCFT) (Figure 1b). Although years of education and age were included as predictors, these demographic variables are not relevant contributors to AD diagnosis. Figure 1c shows the ROC curves for both training and testing datasets for Protocol #1. In the clinical setting, this protocol takes 4 h (240 min) to be completed (Table 3). Overall, this protocol demonstrated excellent ability to distinguish individuals with AD from healthy controls in both the training and testing datasets (Table 3).

The top five predictors identified in Protocol #1 were subsequently used to develop Protocol #2. Consistency analyses revealed that these predictors were robustly selected across multiple data splits, underscoring their reliability (see Figure S2 in the Supplementary Material). Despite including significantly fewer variables than Protocol #1, Protocol #2 maintains strong discriminatory power to differentiate individuals diagnosed with AD from healthy controls (Table 3). Implementing Protocol #2 in the clinical setting drastically reduces the evaluation time from 240 min (4 h) to 25 min (Table 3).

The application of the OneR algorithm identified the neuropsychological variables T51, T53, T1, T19, and T28 as the most accurate predictors of AD diagnosis when used independently (Table 4a). These variables were subsequently included in Protocol #3. Other neuropsychological variables include T27, T25, T43, T23 and T3. Our results demonstrate that the xgbTree ML model, fitted to this protocol with these predictors, effectively distinguished individuals diagnosed with AD from those without the diagnosis while significantly reducing the evaluation time (Protocol #3, Table 3). Compared to the current neuropsychological protocol (Protocol #1), implementing Protocol #3 significantly reduces the evaluation time, on average, to 35 min (Protocol #3, Table 3)

We identified the neuropsychological variables T4, T10, T13, and T16 as those with the highest PPS (Table 2 and Table 4b). In addition to gender and schooling, other relevant neuropsychological variables include T45, T8, T7 and T32 (Table 2 and Table 4b). While significantly reducing the average evaluation time to 25 min for participants, the xgbTree ML model fitted to Protocol #4 performs poorly for differentiating individuals with AD from healthy controls (Table 3).

Overall, the performance metrics based on the xgbTree algorithm showed that Protocol #1, which includes all neuropsychological measurements, achieved the best overall performance. Specifically, its testing accuracy was 0.92, outperforming the other evaluation protocols (Table 3). Similarly, its specificity of 0.90 exceeded the 0.88 and 0.87 observed in Protocols #2 and #3, respectively. Protocol #1 also demonstrated higher AUC values compared to the others (0.92 versus 0.91 for Protocol #2 and 0.90 for Protocol #3). In terms of sensitivity, Protocol #1 scored 0.94, matching the sensitivity values of Protocols #2 and #3. On the other hand, Protocol #4, along with the BTN and RAVLT, consistently performed worse across all metrics compared to Protocols #1, #2, and #3 despite reducing the average evaluation time to only 25 min (Table 3). Although the metrics of Protocol #2 were slightly lower than those of Protocol #1, the difference was minimal, particularly in sensitivity values. However, the reduction in the average evaluation time from 240 min (4 h) to only 25 min makes it a suitable alternative in the clinical setting.

### 3.3. Compactness, Cohesion and Separation of Individuals of by Neuropsychological Protocol

Table 5 presents the compactness and average silhouette coefficients to evaluate the compactness, cohesion, and separation of individuals according to each ML-optimized neuropsychological protocol.

Our results suggest that Protocol #4 provides the greatest compactness of individuals, followed by Protocols #2 and #3. In contrast, the full protocol and those based on specific neuropsychological tests (BNT and RAVLT) do not demonstrate the same level of performance. Meanwhile, Protocol #2, constructed using the top five predictors identified by the xgbTree algorithm, exhibits the highest silhouette coefficient, followed by Protocol #3, RAVLT, Protocol #1, BNT, and Protocol #4. This finding indicates that reducing the full protocol to only the top 5 predictors substantially improve the distribution of individuals within our sample (Table 5).

### 3.4. ML Explainability of the ICN-UN Battery

Our results support that Protocol #2, namely the ICN-UN Neuropsychological Battery, outperforms all other ML-optimized protocols evaluated in this study (Table 3 and Table 5). This protocol comprises the T53 (FAST), T51 (Lawton & Brody Scale), T1 (MMSE) and T29 (Rey Auditory Verbal Memory Recognition, “Yes”) and T19 (Recall in the ROCFT) variables (Table 2 and Table 3).

**Figure 2:**
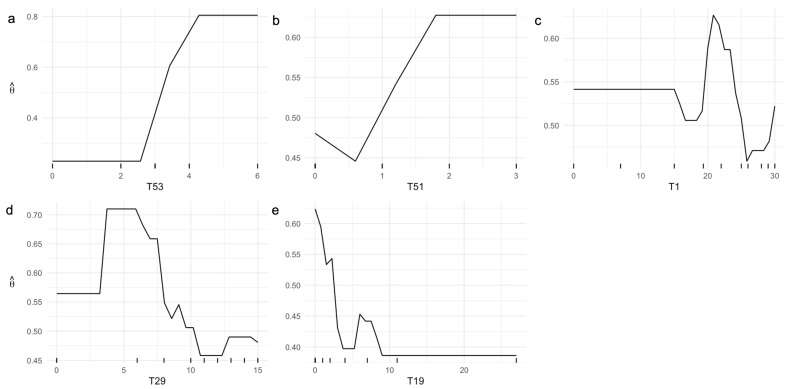
Univariate PDPs illustrating the probability of AD diagnosis, $\hat{\theta }$, as the (**a**) T53, (**b**) T51, (**c**) T1, (**d**) T29, and (**e**) T19 test scores of the ICN-UN Neuropsychological Battery independently vary.

According to the ML-based predictive model for AD status, ML explainability analyses via PDPs reveal notable patterns in the relationship between test scores and the probability of an AD diagnosis (Figure 2). Specifically, having at least four points on test T53 corresponds to a probability of AD diagnosis exceeding 70% (Figure 2a).

Similarly, scoring one or more points on test T51 yields a probability above 50% for a positive AD diagnosis (Figure 2b). Analysis of T1 indicates that scores between 20 and 25 points are associated with the highest probability of an AD diagnosis, whereas scores greater than 25 points reduce this probability (Figure 2c). Furthermore, scores above 10 points on T29 (Figure 2d) or below 3 points on T19 (Figure 2e) are indicative of a negative AD diagnosis.

**Figure 3:**
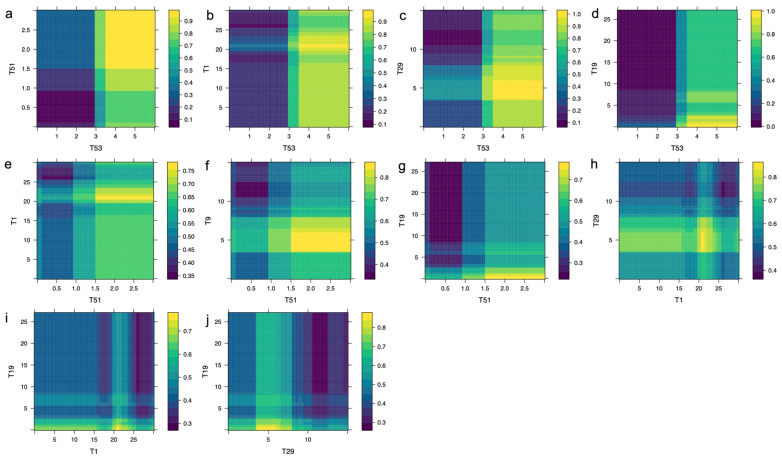
Bivariate PDPs illustrating the probability of AD diagnosis, $\hat{\theta }$, as (**a**) T53 and T51, (**b**) T53 and T1, (**c**) T53 and T29, (**d**) T53 and T19, (**e**) T51 and T1, (**f**) T51 and T9, (**g**) T51 and T19, (**h**) T1 and T29, (**i**) T1 and T19, and (**j**) T29 and T19 neuropsychological tests of the proposed battery simultaneously vary. Higher values of $\hat{\theta }$ are shown in yellow.

Figure 3 demonstrates that the combined effect of specific neuropsychological test scores significantly influences the probability of AD diagnosis. Scores where T53 exceeds 3 combined with T51 greater than 1 produce a probability higher than 0.7 (Figure 3a), and when T53 exceeds 3 alongside T1 scores above 15, the probability increases to over 0.8 (Figure 3b). More stringent combinations, such as T53 greater than 3 with T29 between 3 and 8, yield a probability exceeding 0.9 (Figure 3c). Similarly, T53 > 3 combined with T19 < 3 leads to a probability greater than 0.8 (Figure 3d). Additional combinations involving T51 > 1 with T1 in the 20–25 range (Figure 3e), T51 > 1 with 4 < T9 < 8 (Figure 3f), and T51 > 1 with T19 < 3 (Figure 3g) result in probabilities ranging from 0.7 to 0.8. Furthermore, pairs such as T1 between 20 and 25 with 4 < T29 < 8 (Figure 3h), T1 in the same range with T19 < 4 (Figure 3i), and 4 < T29 < 6 with T19 < 4 (Figure 3j) consistently generate probabilities above 0.75, emphasizing the importance of combined neuropsychological scores in AD diagnostic prediction.

## 4. Discussion

Early and accurate diagnosis of individuals with Alzheimer’s disease (AD) is critical for timely intervention, slowing disease progression, and improving patients’ quality of life [49,50]. However, current diagnostic protocols rely heavily on a combination of neuropsychological assessments and neuroimaging (e.g., MRI, PET scans) [11,15,51], which are both costly and time-consuming [13]. These challenges create significant barriers to widespread adoption, particularly in low- and middle-income countries (LMICs), where healthcare resources are often limited [52]. Therefore, developing streamlined cost-effective screening tools is crucial to improve accessibility and scalability in underserved populations.

In this study, we evaluated streamlined neuropsychological screening protocols for AD diagnosis using data from the Alzheimer’s Disease SGR Study in Barranquilla, Colombia [31]. Our results show that these abbreviated protocols achieve diagnostic accuracy comparable to the comprehensive protocol currently used in clinical practice.

The adoption of simplified protocols can greatly reduce assessment time without compromising precision. Such protocols could improve clinical efficiency, reduce patient and clinician burden, improve patient adherence, and optimize healthcare resource use. Importantly, they support broader and earlier AD detection, particularly in low-income communities where access to healthcare professionals is limited and poverty impedes early AD diagnosis [3]. In that sense, a streamlined assessment protocol aligns with the goals of international organizations to expand representation of these populations in AD research [52].

Our results demonstrate a significant reduction in evaluation time using the ML-optimized ICN-UN battery compared to traditional protocols (Table 3). Specifically, the comprehensive neuropsychological assessment protocol currently used in clinical settings requires approximately 240 min (4 h) to complete. In contrast, the ICN-UN ML-optimized battery, which retains high diagnostic accuracy, reduces the total assessment time to under 25 min—representing a nine-fold reduction in time from start to finish. Other abbreviated protocols tested in our study required between 25 and 35 min (Table 3), but perform poorly in terms of cohesion, compactness and separation of individuals (Table 5 and Figure S1 of the Supplementary Material). The time saved by implementing the streamlined battery translates into significant improvements in clinical efficiency, reduced burden for both patients and clinicians, and enables the assessment of more individuals within the same timeframe. This reduction in evaluation time can be especially beneficial in low-resource settings, where clinical capacity and patient adherence are key barriers to early diagnosis of AD.

The collection of ML algorithms assessed in this study achieved precision and sensitivity rates above 90%, surpassing benchmarks set by other studies about AD detection (see Table S2 of the Supplementary Material) [8,52,53,54]. Those studies evaluated only a limited number of algorithms, leaving it unclear whether other methods might perform better. In contrast, the high accuracy demonstrated by our models indicates that these selected algorithms are highly effective in detecting AD, outperforming the BNT (Table 3). This suggests they hold significant potential as supportive tools for healthcare professionals in clinical settings. According to the performance, compactness, and cohesion and separation performance measures, Protocol #2 (i.e., the ICN-UN Neuropsychological Battery) is recommended to assess AD diagnosis in this population (Table 5).

Key strengths of this study include a wide array of neuropsychological tests that was included, allowing for comprehensive cognitive function assessment. Evaluating multiple machine learning models enabled rigorous comparison and selection of the most accurate protocol for AD detection. Despite these positive implications, the study has some limitations. The sample size, though balanced, may not fully represent the general population, warranting future studies with larger and more diverse samples to validate the generalizability of results. Moreover, the study primarily focused on AD detection, suggesting future research could explore the applicability of the ICN-UN neuropsychological battery to distinguishing AD from other types of dementia or even mild cognitive impairment. Longitudinal studies are recommended to assess the protocol’s predictive validity over time, providing valuable insights into its effectiveness across different disease stages.

Although the findings of this study are promising (i.e., our ML-optimized neuropsychological protocols can achieve high diagnostic accuracy with just five key measures; Table 3), further validation in other populations is needed. The current dataset, derived from a cohort of older adults residing in Barranquilla, Colombia, may not fully capture the ethnic, cultural, and educational heterogeneity of other regions, potentially limiting the generalizability of the protocol. Hence, external validation across varying geographic and sociodemographic contexts is crucial to confirm the model’s efficacy and ensure its applicability in low-resource settings [55], particularly in understudied populations.

## 5. Conclusions

We have developed streamlined neuropsychological assessment protocols for AD detection that achieved high performance metrics in our sample of individuals diagnosed with sporadic AD from the Atlántico Department of Colombia (see Table 3).

Our results suggest that the ML-optimized protocol can offer a more time-efficient alternative to traditional assessments, reducing evaluation time while maintaining comparable diagnostic quality in our sample. This approach has the potential to benefit clinical practice by reducing the time and costs associated with comprehensive neuropsychological assessments, ultimately facilitating earlier and more accessible AD diagnosis. Based on the measures of compactness, cohesion, and separation, Protocol #2 (the ICN-UN Neuropsychological Battery) is the preferred method for assessing AD diagnosis in this population, as it reduced the evaluation time from 240 to 25 min while maintaining an accuracy of 92%, sensitivity of 94%, specificity of 90%, and an AUC of 0.92 (Table 3). This protocol also demonstrated strong compactness, high cohesion and separation, and robust differentiation between individuals with AD and healthy controls (Table 5 and Figure S1 of the Supplementary Material). These results indicate that streamlined protocols can substantially decrease assessment duration without compromising AD diagnostic performance. Implementing this reduced protocol in clinical settings could improve the quality of care and enable more timely and effective interventions for individuals with AD.

Despite these promising results, our study has some limitations. The findings are based on a regional cohort, and broader validation in larger and more diverse populations is necessary to confirm the generalizability and clinical utility of the proposed protocol. Future work should include external validation of the streamlined battery in independent and diverse cohorts, as well as prospective implementation studies to confirm its clinical utility across different healthcare settings.

## Figures and Tables

**Table 1:** Demographic characteristics of individuals included in this study.

Variable	All	AD Diagnosis	Controls	Statistic	*p*
	(*n* = 760)	(*n* = 394)	(*n* = 366)		
	*M* (SD)			*t* (*df*)	
Age (years)	76.7 (7.37)	78.9 (7.51)	74.29 (6.41)	−9.00 (751)	<0.001
Years of education	9.02 (14.1)	8.85 (14.67)	9.21 (13.48)	0.354 (749)	0.723
Gender	*n* (%)			χ^2^ (*df*)	
Female	477 (63.18)	264 (67.01)	213 (59)	4.84 (1)	0.027
Male	278 (36.82)	130 (32.99)	148 (41)		

*n*, sample size; *M*, mean; SD, standard deviation; *df*, degrees of freedom.

**Table 2:** Neuropsychological characteristics of individuals included in this study.

Label	Neuropsychological Test	Cases (*n* = 394)	Controls (*n* = 366)	$\hat{\beta }$ (${SE}_{\hat{\beta }}$)	*p*
T1	Mini-Mental State Examination (MMSE)	16.3 (9.5)	26.9 (3.9)	9.331 (0.558)	<0.0001
T2	Mental Control-Wechsler Memory Scale	1.8 (2.9)	4.7 (3.2)	2.687 (0.219)	<0.0001
	Semantic Verbal Fluency				
T3	Letter “a”	6.1 (4.7)	12.8 (4.4)	5.78 (0.348)	<0.0001
T4	Category loss, letter “a”	0.1 (0.3)	0.1 (0.3)	0.018 (0.021)	0.385
T5	Perseverations, letter “a”	0.4 (1)	0.3 (0.7)	−0.16 (0.068)	0.018
	Semantic Verbal Fluency				
T6	Letter “c”	6.2 (4.2)	11.4 (3.8)	4.635 (0.306)	<0.0001
T7	Category loss, letter “c”	0.1 (0.3)	0 (0.2)	−0.022 (0.021)	0.310
T8	Perseverations, letter “c”	0.6 (1.2)	0.6 (1.2)	−0.064 (0.095)	0.498
	Phonological Verbal Fluency				
T9	Letter “f”	3.7 (3.9)	7.7 (4.8)	3.646 (0.34)	<0.0001
T10	Category loss, letter “f”	0.3 (0.7)	0.1 (0.4)	−0.197 (0.048)	<0.0001
T11	Perseverations, letter “f”	0.3 (0.7)	0.4 (0.7)	0.085 (0.059)	0.14600
	Phonological Verbal Fluency				
T12	Letter “a”	3.2 (3.6)	7.2 (4.5)	3.552 (0.318)	<0.0001
T13	Category loss, letter “a”	0.4 (0.8)	0.3 (0.7)	−0.113 (0.06)	0.060
T14	Perseverations, letter “a”	0.2 (0.6)	0.2 (0.5)	−0.003 (0.042)	0.946
T15	Letter “s”	3.4 (3.9)	7.3 (4.7)	3.51 (0.333)	<0.0001
T16	Category loss, letter “s”	0.4 (0.9)	0.3 (0.6)	−0.133 (0.061)	0.028
T17	Perseverations, letter “s”	0.2 (0.5)	0.3 (0.7)	0.109 (0.046)	0.018
	Rey–Osterrieth Complex Figure Test				
T18	Copy	8.1 (10.9)	22.4 (12.1)	12.22 (0.862)	<0.0001
T19	Recall	1 (2.5)	6.4 (5.5)	4.751 (0.319)	<0.0001
T20	Token Test	16 (10.6)	27.1 (17.6)	9.5 (1.133)	<0.0001
	Rey Auditory Verbal Learning Test				
T21	Attempt #1	1.7 (1.5)	3.3 (1.5)	1.445 (0.114)	<0.0001
T22	Attempt #2	2.4 (1.8)	4.8 (1.8)	2.14 (0.135)	<0.0001
T23	Attempt #3	2.9 (2)	5.7 (2.1)	2.521 (0.154)	<0.0001
T24	Attempt #4	3 (2.2)	6.3 (2.3)	2.964 (0.169)	<0.0001
T25	Attempt #5	3.2 (2.3)	7 (2.5)	3.494 (0.183)	<0.0001
T26	Block of Words	1.3 (1.3)	2.7 (1.4)	1.221 (0.104)	<0.0001
T27	Immediate recall	1.5 (1.8)	4.9 (2.5)	3.126 (0.164)	<0.0001
T28	Delayed recall	0.9 (1.7)	4.4 (2.8)	3.238 (0.179)	<0.0001
	Rey Auditory Verbal Memory Recognition				
T29	Yes	7.3 (5.7)	12.1 (2.8)	4.337 (0.348)	<0.0001
T30	No	7.7 (5.9)	12.8 (3.1)	4.601 (0.373)	<0.0001
	Boston Naming Test (BNT)				
T31	Spontaneous words	19.3 (14.4)	35.1 (12.4)	13.293 (1.021)	<0.0001
T32	Semantic words	1.5 (2.4)	2.5 (3.1)	1.076 (0.219)	<0.0001
T33	Total	20.6 (15.1)	37.6 (12.4)	14.552 (1.053)	<0.0001
	Stroop test				
T34	Words	30.1 (29.2)	61.8 (27)	26.669 (2.195)	<0.0001
T35	Colors	21.1 (20.1)	46.6 (18)	21.428 (1.468)	<0.0001
T36	Words + Colors	9.5 (11.5)	24.3 (13)	12.539 (0.961)	<0.0001
	Trail Making Test (TMT)				
T37	Part A	27.3 (64.2)	98 (80.3)	69.966 (8.182)	<0.0001
T38	Part B	18.9 (73.4)	108.9 (144.4)	93.605 (10.274)	<0.0001
	Symbol Digit Test				
T39	Oral part	3.7 (7.4)	17 (13.6)	11.355 (0.85)	<0.0001
T40	Written part	2.9 (5.7)	13.1 (10.6)	8.635 (0.656)	<0.0001
T41	Continuous Auditory Performance Test	8.8 (6.9)	14 (3.7)	4.355 (0.438)	<0.0001
T42	Benton Visual Retention Test	0.9 (1.3)	3.1 (3)	1.869 (0.179)	<0.0001
T43	Clock Drawing Test	2.9 (3.2)	7.4 (3.4)	4.008 (0.256)	<0.0001
	Wisconsin Card Sorting Test (WCST)				
T44	Categories	0.8 (1.2)	1.8 (1.4)	0.883 (0.099)	<0.0001
T45	Non-perseverative errors	7.4 (10)	10.7 (9.2)	2.564 (0.764)	<0.001
T46	Perseverative errors	18.4 (16.3)	19.3 (11.2)	−0.109 (1.122)	0.923
T47	Correct answers	12.2 (12.9)	19.6 (13.4)	6.073 (1.041)	<0.0001
T48	Memory Disorder Scale	2.1 (2.1)	0.7 (0.9)	−1.323 (0.13)	<0.0001
T49	Yesavage Depression Scale	6.3 (5.9)	2.3 (2.3)	−3.827 (0.354)	<0.0001
T50	Barthel Index	37.6 (18)	49.4 (4.6)	9.648 (1.026)	<0.0001
T51	Lawton & Brody Scale	1.7 (1.2)	0.2 (0.5)	−1.382 (0.067)	<0.0001
T52	Katz Index	1.6 (2.2)	0 (0.3)	−1.311 (0.12)	<0.0001
T53	Functional Assessment Staging (FAST)	3.5 (0.9)	2.3 (0.6)	−1.103 (0.057)	<0.0001

$\hat{\beta }$, estimated linear regression coefficient associated with AD diagnosis; ${SE}_{\hat{\beta }}$, estimated standard error of $\hat{\beta }$; *p*, *p*-value. Correction by gender, age at the beginning of the study and years of education was performed to mitigate the effect of confounding variables.

**Table 3:** Performance of protocols for AD diagnosis in the training and testing datasets.

Protocol (Evaluation Time)	Dataset	Performance Measure								
		Se	Sp	PPV	NPV	FDR	FPR	Accuracy	Lift	AUC
#1	Training	1	1	1	1	0	0	1	1.931	1
(240 min)	Testing	0.941	0.899	0.91	0.933	0.09	0.101	0.921	1.75	0.92
#2	Training	0.906	0.926	0.929	0.902	0.071	0.074	0.916	1.795	0.916
(25 min)	Testing	0.941	0.899	0.91	0.933	0.09	0.101	0.921	1.75	0.920
#3	Training	0.895	0.911	0.915	0.89	0.085	0.089	0.902	1.767	0.903
(35 min)	Testing	0.941	0.872	0.888	0.931	0.112	0.128	0.907	1.708	0.906
#4	Training	0.649	0.7	0.699	0.65	0.301	0.3	0.674	1.35	0.674
(25 min)	Testing	0.72	0.679	0.708	0.692	0.292	0.321	0.7	1.363	0.700
BNT	Training	0.714	0.794	0.788	0.721	0.212	0.206	0.752	1.522	0.754
(25 min)	Testing	0.678	0.826	0.808	0.703	0.192	0.174	0.749	1.555	0.752
RAVLT	Training	0.873	0.86	0.87	0.863	0.13	0.14	0.867	1.68	0.867
(25 min)	Testing	0.89	0.862	0.875	0.879	0.125	0.138	0.877	1.683	0.876

AUC, area under the Receiver Operating Characteristic curve; BNT, Boston Naming Test; FDR, false discovery rate; FPR, false positive rate; NPV, negative predictive value; PPV, positive predictive value; RAVLT, Rey Auditory Verbal Learning test; Se, sensitivity; Sp, specificity.

**Table 4:** Top 10 features for classifying individuals with AD based on (**a**) the OneR algorithm and (**b**) the PPS methods.

**(a)**										
**Rank**	1	2	3	4	5	6	7	8	9	10
**Feature**	T51	T53	T1	T19	T28	T27	T25	T43	T23	T3
**Accuracy**	0.818	0.803	0.788	0.7805	0.7786	0.7767	0.7674	0.7655	0.7617	0.7542
**(b)**										
**Rank**	1	2	3	4	5	6	7	8	9	10
**Feature**	Gender	T4	T10	T13	T16	T45	T8	T7	T32	Schooling
**PPS**	0.058	0.037	0.026	0.026	0.026	0.026	0.018	0.014	0.014	0.009

Rank, lower is better.

**Table 5:** Compactness, average silhouette coefficients and accuracy in the testing dataset for the ML-optimized neuropsychological protocols. Rank is shown in ().

**Protocol**	1	2	3	4	BNT	RAVLT
Compactness	158.869 (6)	10.487 (2)	11.593 (3)	8.83 (1)	22.23 (5)	20.28 (4)
Average silhouette	0.226 (4)	0.371 (1)	0.332 (2)	0.025 (6)	0.197 (5)	0.249 (3)
Accuracy	0.921 (1)	0.921 (1)	0.907 (3)	0.7 (6)	0.749 (5)	0.877 (4)

For Rank, lower is better.

## Data Availability

The data presented in this study are available upon reasonable request from the corresponding authors. They are not publicly available due to the ongoing nature of the study and our commitment to protecting the privacy and confidentiality of our patients.

## Supplementary Materials

The following supporting information can be downloaded at: https://www.mdpi.com/article/10.3390/diagnostics15233045/s1, Table S1. ML algorithms used to predict Alzheimer’s disease (AD) diagnosis. Figure S1. Correlation between (**a**) accuracy and compactness (*r* = 0.372, *p* = 0.467), and (**b**) accuracy and the silhouette coefficient (*r* = 0.856, *p* = 0.029) for the proposed neuropsychological protocols. The ICN-UN Neuropsychological Battery is shown in **green**. For more information see Table 3 in the main manuscript. BNT, Boston Naming Test; RAVLT, Rey Auditory Verbal Learning test. Figure S2. Trace plots illustrating the variable importance scores for predictors included in the ICN-UN Neu-ropsychological Battery (Protocol #2). A robust sampling procedure involving 500 random samples was per-formed based on the xgbTree ML algorithm. For each sample, variable importance was quantified through a cross-validation approach on the training dataset. The mean (M) ± standard deviation (SD) variable importance for the top predictors were as follows: T53: 93.7 ± 11.6, T51: 76.8 ± 25.7, T19: 29.2 ± 9.5, T29: 23.0 ± 4.6, and T1: 25.2 ± 18.9. See Table 1 in the main document for detailed descriptions of the neuropsychological variables. Table S2. Research papers in Web of Science (WoS) assessing the performance of ML algorithms for AD diag-nosis. In this case, the query “Alzheimer’s disease (Title) AND neuropsychology (Topic) AND diagnosis (Topic) AND battery (Topic) and Article (Document Types) and 2020 or 2021 or 2022 or 2023 or 2024 or 2025 (Publication Years) and Article (Document Types)” was used. Research papers assessing the diagnosis of AD using neuropsychological variables and ML-based classification models are highlighted in **green**.

## Abbreviations

The following abbreviations are used in this manuscript:

AD	Alzheimer’s Disease
AUC	Area Under the Receiver Operating Characteristic Curve
BNT	Boston Naming Test
CART	Classification and Regression Trees
FAST	Functional Assessment Staging Test
LMICs	Low- and Middle-Income Countries
ML	Machine Learning
MMSE	Mini-Mental State Examination
MoCA	Montreal Cognitive Assessment
NPV	Negative Predictive Value
PPS	Predictive Power Score
PPV	Positive Predictive Value
RAVLT	Rey Auditory Verbal Learning Test
RF	Random Forest
ROC	Receiver Operating Characteristic
ROCFT	Rey–Osterrieth Complex Figure Test
Se	Sensitivity
Sp	Specificity
SVM	Support Vector Machines
TMT	Trail Making Test
WCST	Wisconsin Card Sorting Test
