# Supplementary material for: The ICN-UN Battery: A Machine Learning-Optimized Tool for Expeditious Alzheimer’s Disease Diagnosis
Source: Diagnostics (Basel). 2025 Nov 28;15(23):3045. doi: 10.3390/diagnostics15233045 (PMC12691277; doi:10.3390/diagnostics15233045)
Supplement: Supplementary file 1 [file diagnostics-15-03045-s001.zip › diagnostics-3904261-supplementary.pdf]

diagnostics

Supplementary Material

The ICN-UN Battery: A Machine Learning-Optimized Tool for Expeditious Alzheimer's Disease Diagnosis

Ernesto Barceló [1,2,3,*], Duban Romero [1], Ricardo Allegri [4], Eliana Meza [1], María I. Mosquera-Heredia [5], Oscar M. Vidal [5], Carlos Silvera-Redondo [5], Mauricio Arcos-Burgos [6], Pilar Garavito-Galofre [5] and Jorge I. Vélez [7,*]

1 Instituto Colombiano de Neuropedagogía, Barranquilla 080020, Colombia; rduban@uninorte.edu.co (D.R.); neurocognitiva@icnweb.org (E.M.)
2 Department of Health Sciences, Universidad de La Costa, Barranquilla 080002, Colombia
3 Grupo Internacional de Investigación Neuro-Conductual (GIINCO), Universidad de La Costa, Barranquilla 080002, Colombia
4 Institute for Neurological Research FLENI, Montañeses 2325, Buenos Aires C1428AQK, Argentina; rallegri@fleni.org.ar
5 Department of Medicine, Universidad del Norte, Barranquilla 081007, Colombia; mosquerai@uninorte.edu.co (M.I.M.-H.); oorjuela@uninorte.edu.co (O.M.V.); csilvera@uninorte.edu.co (C.S.-R.); mpgaravi@uninorte.edu.co (P.G.-G.)
6 Grupo de Investigación en Psiquiatría (GIPSI), Departamento de Psiquiatría, Instituto de Investigaciones Médicas, Facultad de Medicina, Universidad de Antioquia, Medellín 050010, Colombia; mauricio.arcos@udea.edu.co
7 Department of Industrial Engineering, Universidad del Norte, Barranquilla 081007, Colombia
* Correspondence: erbarcelo@yahoo.com (E.B.); jvelezv@uninorte.edu.co (J.I.V.)

Academic Editor: Mohammad Shahadat Hossain

Received: 15 September 2025
Revised: 18 November 2025
Accepted: 22 November 2025
Published: 28 November 2025

Citation: Barceló, E.; Romero, D.; Allegri, R.; Meza, E.; Mosquera-Heredia, M.I.; Vidal, O.M.; Silvera-Redondo, C.; Arcos-Burgos, M.; Garavito-Galofre, P.; Vélez, J.I. The ICN-UN Battery: A Machine Learning-Optimized Tool for Expeditious Alzheimer's Disease Diagnosis. *Diagnostics* **2025**, *15*, 3045. https://doi.org/10.3390/diagnostics15233045

Copyright: © 2025 by the authors. Licensee MDPI, Basel, Switzerland. This article is an open access article distributed under the terms and conditions of the Creative Commons Attribution (CC BY) license (https://creativecommons.org/licenses/by/4.0/).

Table S1. ML algorithms used to predict Alzheimer's disease (AD) diagnosis.

Algorithm	Method[a]	Type	Description
Classification and Regression Tree (CART)	rpart, rpart2, rpart1SE	Classification and Regression	Non-parametric method that recursively splits data into homogeneous subgroups based on predictive variables.
Bagged CART	treebag	Classification and Regression	Combines multiple CART models to improve prediction accuracy and reduce overfitting.
Random Forest (RF)	rf	Classification and Regression	Combines multiple decision trees, using random feature subsets, to improve prediction accuracy and reduce overfitting.
Extreme Gradient Boosting (XGBoost)	xgbTree , xgbLinear	Classification and Regression	Uses gradient boosting to combine multiple decision trees, improving prediction accuracy and reducing overfitting.
Support Vector Machines (SVM)	svmLinear, svmLinear2, svmPoly, svmRadial	Classification and Regression	Classifies data by transforming it into a higher-dimensional space using kernel functions.
Linear Discriminant Analysis (LDA)	lda, lda2	Classification and Regression	Linear method that projects high-dimensional data onto a lower-dimensional space to enhance classification accuracy.
K-nearest neighbors (KNN)	knn	Classification and Regression	Non-parametric method that classifies data based on the majority vote of its nearest neighbors in the feature space.
Generalized Linear Model (GLM)	glm, bayesglm	Regression	Linear model that accommodates non-normal data by employing a link function to map data to a linear space.
Model Averaged Neural Network	avNNet	Classification and Regression	Technique that combines multiple neural network models to enhance prediction accuracy and reduce overfitting.

[a] Refers to the string used to for estimating a particular ML algorithm via the caret package in R. The complete list of ML algorithms implemented in caret is available at https://topepo.github.io/caret/available-models.html.

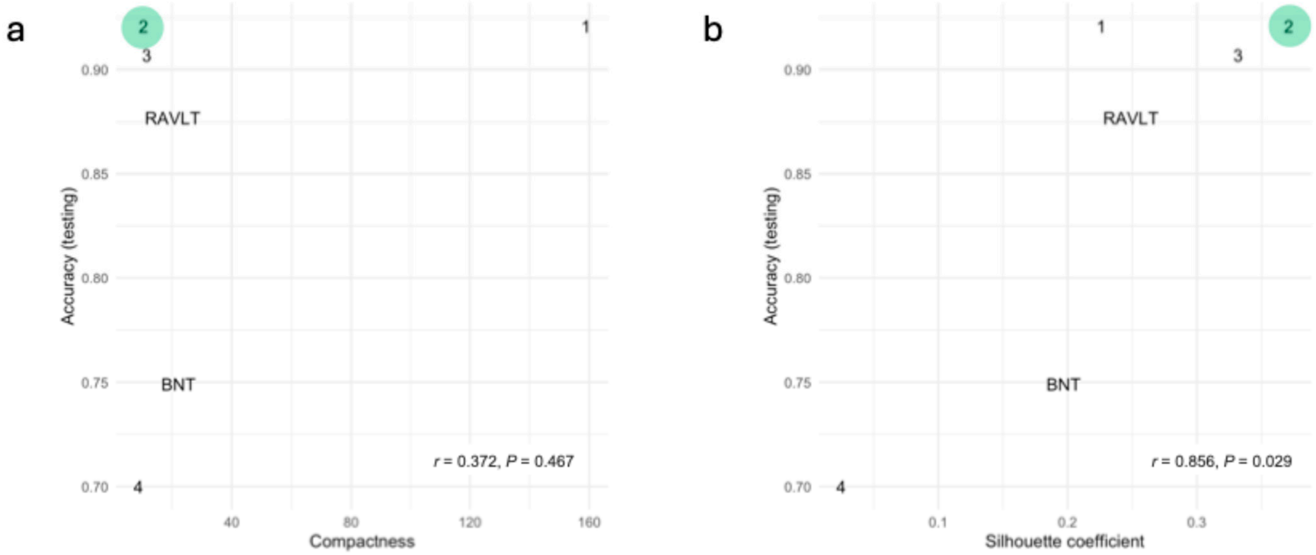

Figure 1S. Correlation between **(a)** accuracy and compactness (r=0.372, P=0.467), and **(b)** accuracy and the silhouette coefficient (r=0.856, P=0.029) for the proposed neuropsychological protocols. The ICN-UN Neuropsychological Battery is shown in green. For more information see Table 3 in the main manuscript. BNT, Boston Naming Test; RAVLT, Rey Auditory Verbal Learning test.

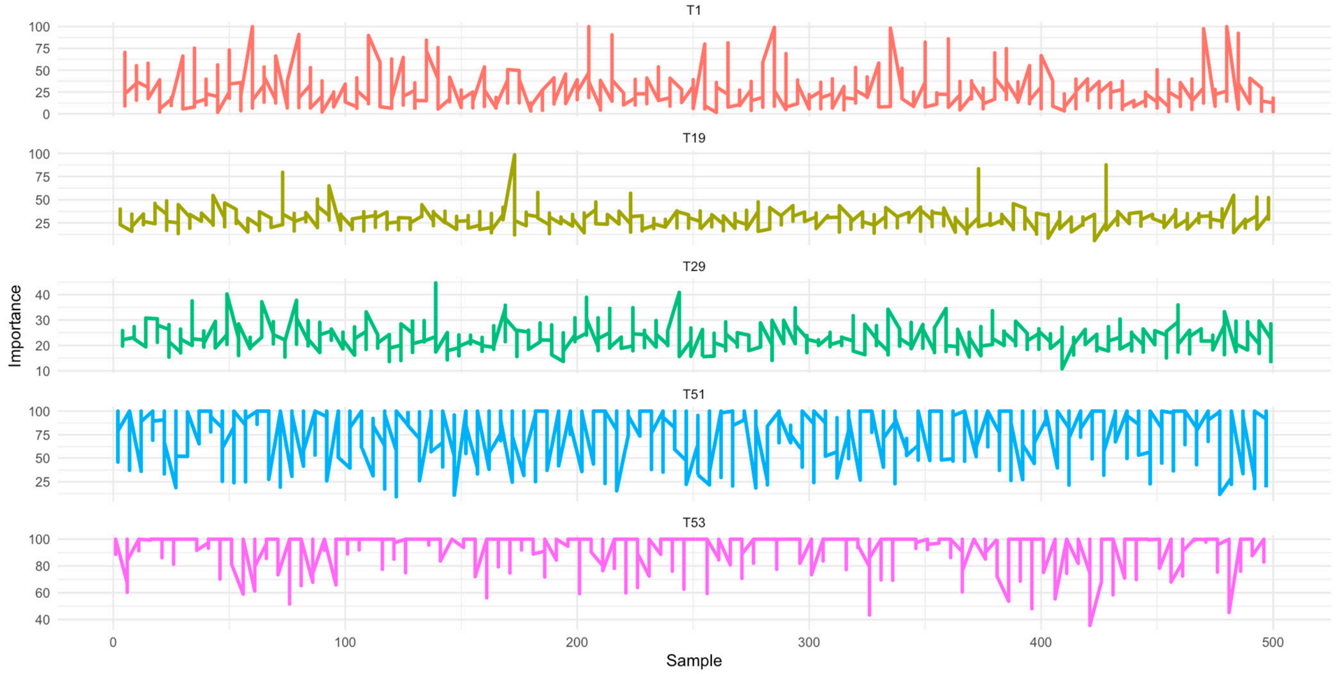

Figure 2S. Trace plots illustrating the variable importance scores for predictors included in the ICN-UN Neuropsychological Battery (Protocol #2). A robust sampling procedure involving 500 random samples was performed based on the xgbTree ML algorithm. For each sample, variable importance was quantified through a cross-validation approach on the training dataset. The mean (*M*) ± standard deviation (SD) variable importance for the top predictors were as follows: T53: 93.7 ± 11.6, T51: 76.8 ± 25.7, T19: 29.2 ± 9.5, T29: 23.0 ± 4.6, and T1: 25.2 ± 18.9. See Table 1 in the main document for detailed descriptions of the neuropsychological variables.

Table S2. Research papers in Web of Science (WoS) assessing the performance of ML algorithms for AD diagnosis. In this case, the query "Alzheimer's disease (Title) AND neuropsychology (Topic) AND diagnosis (Topic) AND battery (Topic) and Article (Document Types) and 2020 or 2021 or 2022 or 2023 or 2024 or 2025 (Publication Years) and Article (Document Types)" was used. Research papers assessing the diagnosis of AD using neuropsychological variables and ML-based classification models are highlighted in **green**.

Summary	Performance Metric Achieved	Reference
Machine learning-based optimization of a neuropsychological battery for AD diagnosis (ICN-UN), with substantial time reduction.	Accuracy: 0.92, Sensitivity: 0.94, Specificity: 0.90, AUC: 0.92	This study
Cognitive profiles in a European cohort predicted biomarker-defined AD status. Supports neuropsychological assessment for preclinical AD identification.	Not provided.	[1]
Developed and validated the BHA-CS, a brief cognitive composite score for early detection and monitoring of AD and related disorders.	At 85% specificity: 100% sensitivity for dementia; 84% sensitivity for MCI (English speakers); 81% sensitivity for MCI/dementia (Spanish speakers).	[2]
The revised Addenbrooke's Cognitive Examination (ACE-R) differentiates dementia with Lewy bodies from AD using a memory /visuospatial ratio	Ratio cut-off of 1.1: Sensitivity 82%, Specificity 68% (DLB vs AD).	[3]
Validated a short, digital spatial memory test as an efficient tool to detect impairment in AD and MCI; rapid and scalable for neuropsychological screening.	Not provided.	[4]
Aggregation of abnormal memory scores in amnestic MCI cases predicts risk of developing AD dementia.	Not provided.	[5]
Neuropsychological profiles in a South American cohort, stratified by amyloid biomarker status, showed distinct cognitive features dependent on biomarker findings; highlights need for population-specific neuropsychological assessment.	Not reported.	[6]
Studied the association between hippocampal volume and performance on African-validated neuropsychological memory tests in adults with probable AD in the DRC.	Diagnostic accuracy not specified.	[7]

Assessed the Spanish English Neuropsychological Assessment Scale (SENAS) in older Latines and high-risk individuals; validated for cross-cultural neuropsychological screening and tracking of early cognitive changes related to AD.	Not stated in abstract.	[8]
Early clinical assessment and brief cognitive/functional tests can predict rapid decline in AD.	Prediction models: AUCs between 0.74 and 0.86 for various outcomes.	[9]
Mapped the sequence of cognitive decline on the NIH-Toolbox battery in Latino adults with autosomal dominant AD.	Explicit diagnostic accuracy not reported.	[10]
Reviewed current practices for MCI due to AD across Asia, highlighted neuropsychological batteries for early diagnosis.	Performance metrics focused on system-level barriers rather than instrument accuracy.	[11]
Four ML classifiers (random forest, SVM, logistic regression, kNN) using neuropsychological subtests and neuropsychiatric symptoms to classify AD vs. behavioral variant frontotemporal dementia (bvFTD).	Accuracy (best model): 0.93; AUC: 0.95	[12]
Random Forest classifiers using cognitive assessment results to classify and predict mild cognitive impairment (MCI) and AD.	Accuracy: 0.87 (AD vs. MCI); Variable importance for test selection shown	[13]
Combined classic and novel neuropsychological tests with ML for AD vs. other diagnoses in 158 patients.	Accuracy (early AD): 0.89; Accuracy (overall): 0.82	[14]

References

1. Terrera, G.M.; Harrison, J.E.; Ritchie, C.W.; Ritchie, K. Cognitive Functions as Predictors of Alzheimer's Disease Biomarker Status in the European Prevention of Alzheimer's Dementia Cohort. *J Alzheimers Dis* 2020, *74*, 1203–1210, doi:10.3233/JAD-191108.
2. Tsoy, E.; Erlhoff, S.J.; Goode, C.A.; Dorsman, K.A.; Kanjanapong, S.; Lindbergh, C.A.; La Joie, R.; Strom, A.; Rabinovici, G.D.; Lanata, S.C.; et al. BHA-CS: A Novel Cognitive Composite for Alzheimer's Disease and Related Disorders. *Alzheimer's and Dementia: Diagnosis, Assessment and Disease Monitoring* 2020, *12*, doi:10.1002/dad2.12042.
3. Prats-Sedano, M.A.; Savulich, G.; Surendranathan, A.; Donaghy, P.C.; Thomas, A.J.; Rowe, J.B.; Su, L.; O'Brien, J.T. The Revised Addenbrooke's Cognitive Examination Can Facilitate Differentiation of Dementia with Lewy Bodies from Alzheimer's Disease. *Int J Geriatr Psychiatry* 2021, *36*, 831–838, doi:10.1002/GPS.5483.

4. Poos, J.M.; van der Ham, I.J.M.; Leeuwis, A.E.; Pijnenburg, Y.A.L.; van der Flier, W.M.; Postma, A. Short Digital Spatial Memory Test Detects Impairment in Alzheimer's Disease and Mild Cognitive Impairment. *Brain Sci* 2021, *11*, doi:10.3390/BRAINSCI11101350.
5. Bradfield, N.I.; Ellis, K.A.; Savage, G.; Maruff, P.; Burnham, S.; Darby, D.; Lautenschlager, N.T.; Martins, R.N.; Masters, C.L.; Rainey-Smith, S.R.; et al. Aggregation of Abnormal Memory Scores and Risk of Incident Alzheimer's Disease Dementia: A Measure of Objective Memory Impairment in Amnestic Mild Cognitive Impairment. *Journal of the International Neuropsychological Society* 2021, *27*, doi:10.1017/S135561772000079X.
6. Clarens, M.F.; Crivelli, L.; Calandri, I.; Chrem Méndez, P.; Martin, M.E.; Russo, M.J.; Campos, J.; Surace, E.; Vázquez, S.; Sevlever, G.; et al. Neuropsychological Profile of Alzheimer's Disease Based on Amyloid Biomarker Findings Results from a South American Cohort. *Applied Neuropsychology:Adult* 2022, *29*, doi:10.1080/23279095.2020.1756816.
7. Ikanga, J.; Hickle, S.; Schwinne, M.; Epenge, E.; Gikelekele, G.; Kavugho, I.; Tsengele, N.; Samuel, M.; Zhao, L.; Qiu, D.; et al. Association Between Hippocampal Volume and African Neuropsychology Memory Tests in Adult Individuals with Probable Alzheimer's Disease in Democratic Republic of Congo. *Journal of Alzheimer's Disease* 2023, *96*, doi:10.3233/JAD-230206.
8. Tureson, K.N.; Beam, C.R.; Medina, L.D.; Segal-Gidan, F.; D'Orazio, L.M.; Chui, H.; Torres, M.; Varma, R.; Ringman, J.M. Use of the Spanish English Neuropsychological Assessment Scale in Older Adult Latines and Those at Risk for Autosomal Dominant Alzheimer's Disease. *J Clin Exp Neuropsychol* 2023, *45*, doi:10.1080/13803395.2023.2284971.
9. Alvarez-Sanchez, L.; Pereto, M.; Garcia-Valles, L.; Balaguer, A.; Pena-Bautista, C.; Ferre-Gonzalez, L.; Baquero, M.; Pericas, C.C. Fast Declining Prediction in Alzheimer's Disease from Early Clinical Assessment. *Curr Neuropharmacol* 2025, *23*, 602–611, doi:10.2174/011570159X332930240925095423.
10. Petkus, A.J.; Sonti, A.N.; Montoya, L.; Rowe, B.; Sagare, A.; Ringman, J.M. Sequence of Decline on the NIH-Toolbox Cognitive Battery in a Predominantly Latino Sample with Autosomal Dominant Alzheimer's Disease. *J Alzheimers Dis* 2025, *103*, 1150–1160, doi:10.1177/13872877241312934.
11. Choi, S.H.; Kim, S.Y.; Ong, P.A.; Chin, A.V.; Dominguez, J.; Chen, C.L.H.; Senanarong, V.; Hu, C.J.; Tripathi, M.; Mok, V.; et al. The Patient Pathway for Mild Cognitive Impairment Due to Alzheimer's Disease in Asia: Current Practices, Barriers, and Expert Recommendations for Optimization. *J Prev Alzheimers Dis* 2025, *12*, 100215, doi:10.1016/j.tjpad.2025.100215.
12. Goodwin, G.J.; Fonseca, J.; Mehrzad, S.; Cummings, J.L.; John, S.E. Classification of AD and BvFTD Using Neuropsychological and Neuropsychiatric Variables: A Machine Learning Study. *Alzheimer's & Dementia* 2025, *21*, e70782, doi:10.1002/ALZ.70782.
13. Cazzolli, C.; Chierici, M.; Dallabona, M.; Guella, C.; Jurman, G. Neuropsychological Tests and Machine Learning: Identifying Predictors of MCI and Dementia Progression. *Aging Clin Exp Res* 2025, *37*, 79, doi:10.1007/S40520-025-02962-4.
14. Gurevich, P.; Stuke, H.; Kastrup, A.; Stuke, H.; Hildebrandt, H. Neuropsychological Testing and Machine Learning Distinguish Alzheimer's Disease from Other Causes for Cognitive Impairment. *Front Aging Neurosci* 2017, *9*, doi:10.3389/FNAGI.2017.00114.

Disclaimer/Publisher's Note: The statements, opinions and data contained in all publications are solely those of the individual author(s) and contributor(s) and not of MDPI and/or the editor(s). MDPI and/or the editor(s) disclaim responsibility for any injury to people or property resulting from any ideas, methods, instructions or products referred to in the content.
